# CXorf48 is a potential therapeutic target for achieving treatment-free remission in CML patients

**DOI:** 10.1038/bcj.2017.84

**Published:** 2017-09-01

**Authors:** M Matsushita, K Ozawa, T Suzuki, M Nakamura, N Nakano, S Kanchi, D Ichikawa, E Matsuki, M Sakurai, D Karigane, H Kasahara, N Tsukamoto, T Shimizu, T Mori, H Nakajima, S Okamoto, Y Kawakami, Y Hattori

**Affiliations:** 1 Division of Clinical Physiology and Therapeutics, Keio University, Faculty of Pharmacy, Shiabakoen, Minato-ku, Tokyo, Japan; 2 Division of Hematology, Department of Medicine, Keio University School of Medicine, Shinjuku-ku, Tokyo, Japan; 3 Division of Cellular Signaling, Institute for Advanced Medical Research, Keio University, School of Medicine, Shinjuku-ku, Tokyo, Japan; 4 Department of Stem Cell and Immune Regulation, Yokohama City University Graduate School of Medicine, Kanazawa-ku, Yokohama, Japan

## Abstract

Although the introduction of tyrosine kinase inhibitors (TKIs) has improved overall survival of patients with chronic myeloid leukemia (CML), about half of the patients eventually relapse after cessation of TKIs. In contrast, the remainder of the patients maintain molecular remission without TKIs, indicating that the patients’ immune system could control proliferation of TKI-resistant leukemic stem cells (LSCs). However, the precise mechanism of immunity against CML-LSCs is not fully understood. We have identified a novel immune target, CXorf48, expressed in LSCs of CML patients. Cytotoxic T cells (CTLs) induced by the epitope peptide derived from CXorf48 recognized CD34^+^CD38^−^ cells obtained from the bone marrow of CML patients. We detected CXorf48-specific CTLs in the peripheral blood mononuclear cells from CML patients who have discontinued imatinib after maintaining complete molecular remission for more than 2 years. Significantly, the relapse rate of CXorf48-specific CTL-negative patients was 63.6%, compared to 0% in CXorf48-specific CTL-positive patients. These results indicate that CXorf48 could be a promising therapeutic target of LSCs for immunotherapy to obtain durable treatment-free remission in CML patients.

## Introduction

Recent studies reported that long-term administration of tyrosine kinase inhibitors (TKIs) increased economic burden and adverse events, including vascular events and impaired quality of life in patients with chronic myeloid leukemia (CML).^1, 2, 3^ To resolve these problems, the feasibility of discontinuation of TKIs after sustained complete molecular response (CMR) has been evaluated in several clinical trials; these trials have shown that about half of patients can maintain CMR after the discontinuation of TKIs, which means treatment-free remission (TFR).^4, 5, 6^ Indeed, TFR is now considered as the next therapeutic goal of CML after achieving CMR.^7, 8^

In patients achieving TFR, the immune system is thought to play a role in controlling proliferation of TKI-resistant leukemic stem cells (LSCs) or keeping LSCs quiscent.^9, 10, 11^ Therefore, immunotherapy directly targeting LSCs may be a promising strategy for achieving TFR. However, the detail of T-cell immunity against CML-LSCs in TFR setting is still unknown. Cytotoxic T lymphocytes (CTLs) recognize specific epitope peptides presented by certain human leukocyte antigen (HLA) molecules on the surface of LSCs.^12^ Thus, we tried to find novel LSC antigen peptide recognized by CTLs in the present study. By screening genes highly expressed in cancer cells, we have found *CXorf48* gene. CXorf48 was previously reported to be expressed in several cancers, but not in normal tissues, except for the testes and referred as a candidate cancer-testis antigen only from its expression pattern.^13^ However, expression of CXorf48 in leukemic cells is still undetermined. Moreover, T-cell epitopes derived from this antigen remain unknown. In the present study, we identified the first epitope peptide and investigated whether CXorf48 could be a therapeutic target as LSC antigen in CML patients, particularly in the context of TFR.

## Materials and methods

### Cell lines

Cancer cell lines K562, KU812, KG1a, THP-1, BALL1 and PL21 were obtained from the Health Science Research Resources Bank (National Institute of Biomedical Innovation, Osaka, Japan). The HL60, U937, HEL, Molt4 and Daudi were obtained from American Type Culture Collection (ATCC, Manassas, VA, USA). KMS21 was kindly gifted by Dr Ohtsuki of Kawasaki Medical School, Kurashiki, Japan. These cells were maintained at 37 °C in a humidified atmosphere of 5% CO_2_ in RPMI-1640 medium (Sigma-Aldrich, St Louis, MO, USA) and 10% fetal bovine serum (Invitrogen, Life Technologies, Grand Island, NY, USA).

### Patient samples

To examine the expression of *CXorf48* gene, peripheral blood samples from healthy donors (HD) and CML patients and bone marrow (BM) samples from CML patients were obtained after informed consent. Peripheral blood was also obtained from HLA-A*24:02-positive patients enrolled in Keio STIM trial^14^ at least three time points, which were the day before cessation of imatinib, the third month and sixth month after cessation. Written informed consents were obtained from all HDs and patients. Mononuclear cells were isolated by Ficoll density gradient centrifugation and cryopreserved in Cell Banker reagent (Juji-Field, Tokyo, Japan) in liquid nitrogen until further use. Bone marrow mononuclear cells (BMMNC) from HDs were purchased from AllCells, LLC. (Alameda, CA, USA). This study was approved by the ethics committee of Keio University School of Medicine (20100186, 20140223) and Keio University Faculty of Pharmacy (140425-2, 160715-3).

### Expression of *CXorf48* gene in CML patients with various clinical stages

*CXorf48* gene expression levels in CML cells from patients in the chronic phase, accelerated phase and blastic crisis were obtained from a public repository (Gene Expression Omnibus, accession: GSE4170). Expression levels of the *CXorf48* gene in each group were compared using SPSS statistics version 23 software (IBM Corp., Armonk, NY, USA).

### Sorting of LSCs

Leukemic cells were washed with phosphate-buffered saline (PBS; Sigma-Aldrich) and stained with R-phycoerythrin-conjugated monoclonal antibody against CD34 and fluorescein isothiocyanate-conjugated monoclonal antibody against CD38 (clone AC136,1B6; Miltenyi Biotec, Auburn, CA, USA) for 30 min on ice. After washing with PBS, cells were analyzed and separated using a MoFlo cell sorter system (Beckman Coulter Inc., Brea, CA, USA).

### Detection of *CXorf48* expression with RT–PCR

Expression of the *CXorf48* gene was detected by standard reverse transcription polymerase chain reaction (RT–PCR) or quantitative PCR. Total RNA was extracted from cancer cell lines, BMMNC from patients or HD, and peripheral blood mononuclear cells (PBMNC) from HD using Isogen (Nippon Gene Co. Ltd, Tokyo, Japan). Complementary DNA was synthesized from 1 μg of the total RNA using ReverTra Ace qPCR RT Master Mix with gDNA Remover (TOYOBO, Osaka, Japan), followed by PCRs with *CXorf48* primers (Forward; 5′-gttgtgcctcgccatctttatg-3′, Reverse; 5′-tgcactggggtgatagaaatcg-3′) or *GAPDH* primers (Forward; 5′-tgaacgggaagctcactgg-3′, Reverse; 5′-tccaccaccctgttgctgta-3′) with Taq polymerase (Takara Bio, Shiga, Japan). Quantitative PCR was performed using SsoFast probes supermix (Bio-Rad, Hercules, CA, USA) with *CXorf48* primers and probe (TaqMan Gene Expression Assays; Applied Biosystems, CA, USA) or *GAPDH* primers (Forward; 5′-gacctgacctgccgtctagaaa-3′, Reverse; 5′-cctgcttcaccaccttcttga-3′) and probe (5′-(6-FAM)-acctgccaaatatgatgac-(BHQ-2)-3′). Complementary DNA from K562 cell line was used to make standard curves. Relative gene expression level was calculated as follows: *CXorf48* expression level (sample)/*GAPDH* expression level (sample).

### Immunohistochemical staining

Immunohistochemical staining was performed as follows: Cells were attached to a slide using a Cytospin 4 cytocentrifuge (Thermo Fisher Scientific, Waltham, MA, USA), then fixed with 2% paraformaldehyde and permeabilized with Triton X, followed by blocking with 1% bovine serum albumin in PBS. The sample was stained with mouse anti-CXorf48 antibody (Sigma-Aldrich) followed by Alexa Fluor488 goat-anti-mouse IgG (Molecular Probes, Thermo Fisher Scientific, Eugene, OR, USA).

### Synthetic peptides

CXorf48-derived peptides were identified using the Bioinformatics and Molecular Analysis Section (BIMAS) program (http://bismas.dcrt.nih.gov/molbio/hla_bind/index.html), and the SYPFPEITHI program (http://www.syfpeithi.de/). HLA-A*24:02 binding peptides were synthesized and purified to>98% by HPLC (Sigma-Aldrich Japan, Tokyo, Japan). Purified peptides were dissolved in dimethylsulphoxide and stored in aliquots at −80 °C.

### Generation of CXorf48 peptide-specific CTLs from human PBMCs

Cytotoxic T lymphocytes were generated by *in vitro* stimulation with peptide-pulsed autologous dendritic cells and phytohemagglutinin blasts as described before.^15^ Briefly, PBMNCs were isolated from whole blood of HLA-A*24:02-positive HD by Ficoll density gradient centrifugation. Dendritic cells were generated by isolating CD14^+^ cells using a MACS separation system (Miltenyi Biotec, Bergisch Gladbach, Germany). The CD14^+^ cells were cultured in AIM-V medium supplemented with 10% human serum, 100 ng/ml of IL-4 (R&D Systems, Minneapolis, MN, USA) and 100 ng/ml of GM-CSF (R&D Systems) for 5 days, then 20 ng/ml of TNF-α (R&D Systems) was added to generate dendritic cells. Phytohemagglutinin blasts were derived from CD14^−^ cells by culturing in AIM-V medium supplemented with 10% human serum, 100 units of IL-2 and 1 μg/ml of phytohemagglutinin for 2 days. The dendritic cells or phytohemagglutinin blasts were pulsed with 50 μg/ml of peptide at room temperature for 3 h and then irradiated. On day 0, CD14^−^ cells were stimulated with irradiated peptide-pulsed dendritic cells in AIM-V medium with 10% human serum supplemented with 10 ng/ml of IL-7 (R&D Systems), then 50 ng/ml of IL-2 was added every 2–3 days. The cells were stimulated weekly with irradiated peptide-pulsed autologous phytohemagglutinin blasts, then CD3^+^CD8^+^ cells were purified with immunomagnetic beads (Miltenyi Biotec) and used for enzyme-linked immunospot assay or cytotoxicity assay.

### Enzyme-linked immunospot assay

IFN-γ secretion of CTLs in response to target cells was detected using enzyme-linked immunospot assay. Briefly, effector cells were incubated with target cells in plates coated with anti-IFNγ antibody (1-D1K; Mabtech Inc., Cincinnati, OH, USA). After incubation for 20 h, biotinylated antibody specific for IFN γ (7-B6-1; Mabtech) was added for 2 h at room temperature and then streptavidin-alkaline phosphatase (Mabtech) was added for 1 h. To develop spots, nitroblue tetrazolium and 5-bromo-4-chloro-3-indolyl phosphate (Bio-Rad) were added and the color development was stopped by rinsing with distilled water. The resulting spots were counted using a CTL-ImmunoSpot analyzer (Cellular Technology Ltd., Shaker Heights, OH, USA).

### Cytotoxicity assay

The cytotoxic activity of the CTLs was measured using a standard ^51^Cr release assay. Target cells were labeled with 50 μCi of ^51^Cr (PerkinElmer Inc. Waltham, MA, USA) for 60 min at 37 °C. CIR-A24 cells were then incubated with 5 μg/ml of peptide for 1 h. The target cells were mixed with effector cells. After 4 h, supernatants were transferred to LumaPlates (PerkinElmer) and allowed to air dry in a hood overnight. Plates were sealed and counted in MicroBeta Trilux instrument (PerkinElmer). Percent specific lysis for ^51^Cr release was calculated as [(experimental ^51^Cr release – spontaneous ^51^Cr release)/(maximum ^51^Cr release – spontaneous ^51^Cr release)] × 100.

### CD107a assay

CD107a assay was performed using IMMUNOCYTO CD107a Detection Kit (MBL, Nagoya, Japan) according to the manufacturer’s instruction. Briefly, 3.0 × 10^4^ cells/well of effector cells were incubated with 3.0 × 10^4^ cells/well of target cells in the presence of fluorescein isothiocyanate-conjugated anti-CD107a antibody and 2 μl of Monensin for 4 h. Then cells were washed with PBS containing 0.5% FBS, and stained with PerCP-conjugated anti-CD3 antibody (Immunostep, Salamanca, Spain), fluorescein isothiocyanate-conjugated anti-CD8 antibody (Miltenyi Biotec). The positivity of CD107a was analyzed on a flow cytometer (LSR II, BD Biosciences, San Jose, CA, USA).

### Dextramer staining of STIM patients samples

The frozen PBMNCs were thawed and washed in CTL medium (CTL), suspended in AIM V 10% AB human serum (LONZA, Basel, Switzerland). CXorf48 peptide (10 μg/ml) and 10 ng/ml of IL-7 were added. IL-2 (10 U/ml) was added every three days. On day 7, CXorf48 peptide and IL-7 were added again and cultured in the presence of IL-2. On day 17, cells were washed with PBS (Sigma), stained with antibodies against CD3 (Immunostep), CD8 (Miltenyi Biotec) and dextramer specific for HIV-derived peptide or CXorf48-derived peptide (Immudex, Copenhagen, Denmark). Those cells were analyzed on a flow cytometer (LSR II, BD Biosciences).

### Statistical analysis

Results are presented as mean±s.e.m. Groups were compared using Student’s *t*-test. For evaluating two-way contingency table, chi-square test was used. Differences were considered significant when *P*-values were less than 0.05.

## Results

### *CXorf48* gene was expressed in various kind of hematological malignancies especially in CML

**Figure 1:**
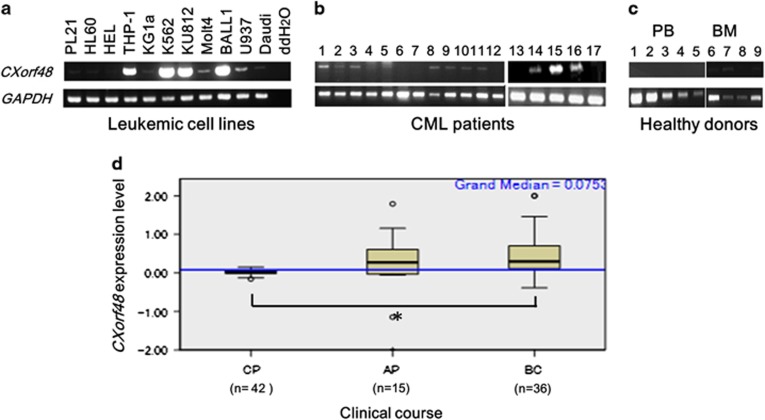
Expression profile of *CXorf48* gene in leukemia cells. *CXorf48* gene expression was detected by conventional RT–PCR using *CXorf48* gene-specific or *GAPDH* gene-specific primers. (**a**) Expression of *CXorf48* gene in cell lines of hematological malignancy. (**b**) Expression of *CXorf48* gene in bone marrow mononuclear cells (BMMNCs) from CML patients at diagnosis. (**c**) Expression of *CXorf48* gene in peripheral blood mononuclear cells (PBMNCs) or BMMNCs from healthy donors (HD). (**d**) *CXorf48* gene expression levels in CML cells from patients in the chronic phase (CP), accelerated phase (AP) and blastic crisis (BC) obtained from a public repository (Gene Expression Omnibus, accession: GSE4170). *P<0.05 (median test).

We evaluated *CXorf48* gene expression in cell lines of various types of hematological malignancies by conventional RT–PCR. Some of leukemic cells including THP-1 (acute myeloid leukemia), K562 (CML), KU812 (CML) and BALL1 (acute lymphocytic leukemia) expressed the gene at high levels (Figure 1a). *CXorf48* gene expression was also detected in 12 out of 17 (70%) of BMMNC samples from CML patients at diagnosis (Figure 1b).

In contrast, *CXorf48* gene expression was not observed in PBMNCs or BMMNCs from HDs, except for one donor (BM7) whose cells showed weak expression (Figure 1c). We also confirmed *CXorf48* expression in CML patients in the chronic phase, accelerated phase and blastic crisis using gene expression profile in samples from patients with CML obtained from a public repository. Gene expression was highest in BC compared to chronic phase (*P*<0.01; Figure 1d).

### *CXorf48* gene was expressed in leukemic stem cells of CML

**Figure 2:**
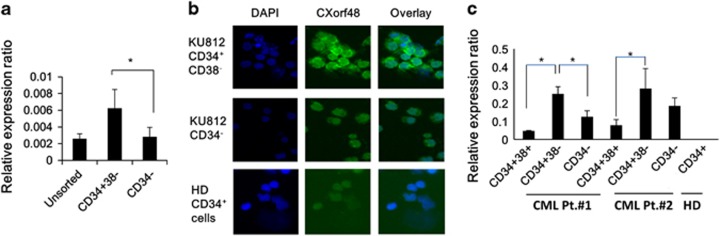
Expression of CXorf48 in CML stem cells. (**a**) *CXorf48* gene expression in unsorted samples, CD34^+^38^−^ fraction, and CD34^−^ fraction of KU812 cells were assessed by quantitative RT–PCR. (**b**) CXorf48 protein expression in sorted KU812 cells or CD34^+^ BMMNCs from healthy donor (HD) was detected by immunohistochemistry using anti-CXorf48 polyclonal antibody. (**c**) *CXorf48* gene expression in CD34^+^38^+^ fraction, CD34^+^38^−^ fraction and CD34^−^ fraction from PBMNCs (CML patient No.1) and BMMNCs (CML patient No.2) was evaluated by quantitative RT–PCR.

To further assess the expression of CXorf48 in LSCs, we sorted the CD34^+^CD38^−^ cells from the CML cell line KU812, since LSCs of myeloid leukemia are considered to be enriched in this fraction.^16, 17^ The results of quantitative PCR showed that CD34^+^38^−^ cells isolated from the CML cells, KU812, expressed higher level of *CXorf48* gene compared to CD34^−^ cells (Figure 2a). Expression of CXorf48 protein in these fractions was also detected by immunohistochemistry. On the other hand, CD34^+^ cells from HD did not express CXorf48 protein (Figure 2b).

We then examined *CXorf48* gene expression in LSCs from two CML patients at diagnosis. In both patients, *CXorf48* gene was expressed higher in CD34^+^38^−^ cells compared to CD34^+^38^+^ cells or CD34^−^ cells. In addition, CD34^+^ cells from HD did not express *CXorf48* gene (Figure 2c).

### Anti-CXorf48 CTLs induced with epitope peptide showed antigen-specific recognition

**Figure 3:**
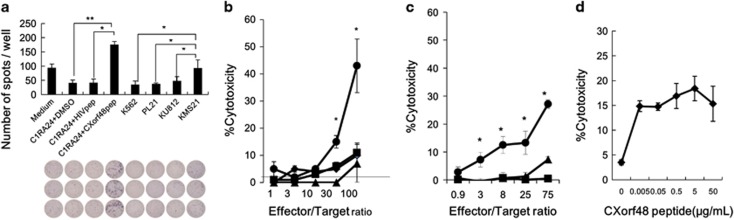
Target recognition by CXorf48-specific CTLs. Antigen specificity of CXorf48-specific CTLs was analyzed. (**a**) IFN-γ secretion was measured from CTLs responding to CIR-A24 cells pulsed with CXorf48^49–57^ peptide or HIV-derived peptide. K562 (HLA-A*24:02^−^ and CXorf48^+^), PL21 (HLA-A*24:02^+^ and CXorf48^−^), KU812 (HLA-A*24:02^−^ and CXorf48^+^), and KMS21 (HLA-A*24:02^+^ and CXorf48^+^) cells were also used as targets. (**b**) The cytotoxicity of CXorf48-specific CTLs against CIR-A24 cells pulsed with CXorf48^49–57^ peptide (•), HIV-derived peptide (▪) or DMSO (▴) was examined by ^51^Cr release assays. (**c**) The cytotoxicity of CXorf48-specific CTLs against cancer cell lines K562 (HLA-A*24:02^−^ and CXorf48^+^;▴), PL21 (HLA-A*24:02^+^ and CXorf48^−^;▪), and KMS21 (HLA-A*24:02^+^ and CXorf48^+^;•) was assessed by ^51^Cr release assays. (**d**) The cytotoxicity of CXorf48-specific CTLs against CIR-A24 cells pulsed with various concentration (0–50 μg/ml) of CXorf48^49–57^ peptide were measured by ^51^Cr release assays. Data are representative of three independent experiments. Each experiment was performed in triplicate. *P<0.05, ** *P*<0.01 (Student’s *t*-test). DMSO, dimethylsulphoxide.

We synthesized five of CXorf48-derived candidate peptides binding to HLA-A*24:02 (most popular HLA-classI allele in Japanese population) based on scores predicted by the SYFPEITHI and BIMAS programs (Supplementary Table 1). Enzyme-linked immunospot assay revealed that the CTL induced with CXorf48^49–57^ peptide (DYGMIDESI) recognized CIR-A24 cells pulsed with this peptide, or HLA-A*24:02-positive and CXorf48-positive KMS21 cells (Figure 3a). Cytotoxic assay also revealed that the CTLs could lyse C1R-A24 cells only when the target cells were pulsed with CXorf48^49–57^peptide (Figure 3b). At the same time, the CTLs lysed only HLA-A*24:02-positive and CXorf48-positive KMS21 cells. HLA-A*24:02-positive and CXorf48-negative PL-21 cells nor HLA-A*24:02-negative and CXorf48-positive K562 cells were lysed by the CTLs (Figure 3c). The recognition by CTLs was dependent on dose of the peptide (Figure 3d).

### Anti-CXorf48 CTLs effectively recognized LSCs from CML patients

**Figure 4:**
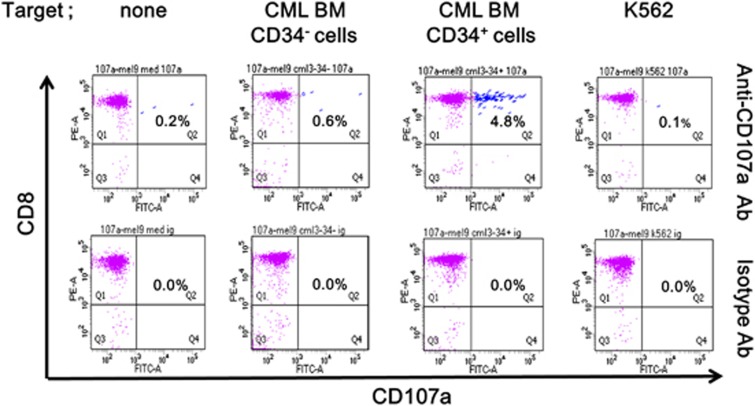
Recognition of patients’ CML cells by CXorf48-specific CTLs. CD34^+^ cells and CD34^−^ cells separated from PBMNCs of CML patient #1 in Figure 2c were incubated with CXorf48-specific CTLs for 4 h in the presence of fluorescein isothiocyanate-labeled anti-CD107 antibodies. The cells were then stained with PerCP-labeled anti-CD3 and anti-CD8 antibodies. Surface expression of CD107a in CD3-positive and CD8-positive fraction was detected by flow cytometry. An isotype antibody was used as a negative control for the anti-CD107a antibody. Percentages indicate the proportion of CD8^+^CD107a^+^ cells in CD3^+^ cells.

We conducted CD107a assay to investigate whether CXorf48-specific CTLs could recognize patient-derived CML-LSCs. Figure 4 showed that 4.8% of CD3^+^CD8^+^ cells expanded by CXorf48^49–57^ peptide highly expressed CD107a when incubated with CD34^+^ BMMNCs from CML patients. Those CTLs did not recognize CD34^−^ BMMNCs from the same donor nor K562 cells.

### CXorf48-specific CTLs were detected in CML patients after imatinib withdrawal

**Figure 5:**
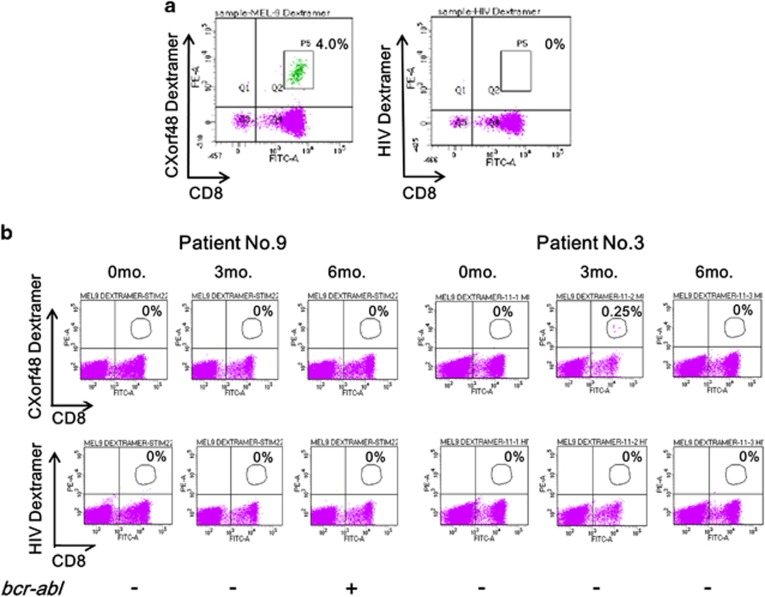
Detection of CXorf48-specific CTLs in peripheral blood of CML patients after discontinuation of imatinib. (**a**) CXorf48-specific CTLs induced by CXorf48^49–57^ peptide were stained with dextramer containing complex of HLA-A*24:02 and CXorf48^49–57^ peptide and analyzed by flow cytometer (left). Dextramer containing complex of HLA-A*24:02 and HIV-derived peptide was used as a negative control (right). (**b**) PBMNCs were isolated from CML patients enrolled in Keio-STIM trial at 0, 3 or 6 months after cessation of imatinib, stained with both FITC-labeled anti-CD8 antibodies and PE-labeled dextramers specific for CXorf48 or HIV and then analyzed by flow cytometry. Percentages of CD3^+^CD8^+^dextramer^+^ cells in CD3^+^ cells are indicated. Positivity of *BCR-ABL1* PCR is indicated below (+: positive, −: negative).

To evaluate immune reactions against CXorf48 in patients with CML, we detected CXorf48-specific CTLs in PBMNCs from the patients. For this purpose, we used CXorf48-specific dextramer which could bind to CTLs recognizing CXorf48^49–57^peptide in HLA-A*24:02-restricted manner. First, we confirmed that specificity of this dextramer by staining anti-CXorf48 CTLs induced from HD’s PBMNCs. Figure 5a shows that 4% of bulk culture of anti-CXorf48 CTLs were positive for CXorf48-specific dextramer, but those cells were not stained with control HIV-specific dextramer. Then we stained PBMNCs from CML patients in whom imatinib was discontinued after remaining in CMR for more than 2 years in KEIO STIM trial.^14^ We used PBMNCs obtained at 0, 3 and 6 months after the cessation of imatinib, since most of molecular relapse occur during this period. Patients’ clinical outcomes were followed up for 29–70 months (median; 49 months) after the cessation for imatinib (Table 1). Figure 5b shows the dextramer staining of a patient who showed molecular relapse at the sixth month after cessation of imatinib (patient no. 9) and a patient who has been remaining in CMR (patient no. 3). We could not detect any CXorf48-specific CTLs in the former patient. On the other hand, CXorf48-specific CTLs were detected in PBMNCs obtained from patient no. 3 in the third month from drug cessation (Figure 5b). In total, we could detect CXorf48-specific CTLs in three out of seven patients (43%) who had maintained CMR at least 37 months after cessation of imatinib (Table 1). On the other hand, we could detect CXorf48-specific CTLs in none of seven patients who had molecular relapse. The relapse rate of CXorf48-specific CTL-negative patients was 63.6%, as compared to 0% in CXorf48-CTL-positive patients. We also tested these results by contingency table made from Table 1 and found that existence of anti-CXorf48 CTL tended to be correlated with maintenance of CMR after cessation of imatinib (*P*=0.051; Supplementary Table 2).

## Discussion

Immunotherapy against LSC-specific antigens is one of the promising strategies to achieve a cure for CML. For this purpose, identification of novel target antigens expressed in LSCs is necessary.

CXorf48 has been reported as a cancer-testis antigen that is expressed in several cancer cell types such as lung cancer, gastric cancer and cervical cancer.^13^ However, its expression in hematological malignancies was not known. Therefore, we first evaluated *CXorf48* gene expression in various leukemic cell lines and determined that CML cells expressed high levels of the *CXorf48* gene. Moreover, the *CXorf48* gene was detected in 70% of BMMNCs from CML patients at diagnosis while PBMNCs or most BMMNCs from HDs did not express this gene. We also confirmed *CXorf48* expression was highest in BC patients using the gene expression profile of CML patients from a public repository. Since it has been reported that CXorf48 enhances the growth of cancer cells by binding to the BRCA1 gene,^18^ this gene could also be related to the disease progression of CML patients.

We next examined CXorf48 expression in CML-LSCs. CD34^+^CD38^−^ cells expressed the *CXorf48* gene more than CD34^−^ cells isolated from the KU812 cell line or CML patients’ samples. CXorf48 protein was also detected in CD34^+^CD38^−^ cells of the KU812 cell line. In contrast, CD34^+^ BM cells from a HD did not express CXorf48. These data suggest that CXorf48 could be a promising LSC target.

Furthermore, we identified the HLA-A*24:02-restricted CXorf48^49–57^ peptide (DYGMIDESI) from five candidate peptides. Enzyme-linked immunospot and cytotoxic assays both indicated that the CTLs induced by this epitope peptide recognized HLA-A*24:02-positive CIR-A24 cells pulsed with the peptide or HLA-A*24:02-positive and CXorf48-positive KMS21 cells. Importantly, these CTLs recognized CD34^+^ cells from BMMNCs of patients with CML but did not recognize CD34^−^ BMMNCs from the same patient. This suggests that CXorf48-specific T cells could preferentially recognize CXorf48-positive cells enriched in CD34^+^ CML cells.

Finally, we analyzed PBMNCs from a series of CML patients who discontinued imatinib after sustaining CMR for more than 24 months in the Keio STIM trial, and the results indicated that CTLs against CXorf48 might play a role in the maintenance of CMR in these patients. By dextramer staining, CXorf48-specific CTLs were found in three patients during the first 6 months after imatinib withdrawal, all of whom have maintained CMR for more than 36 months. In contrast, CXorf48-specific CTLs could not be detected in any patients who relapsed after cessation of imatinib. Thus, CXorf48-specific T cells may play a role in the control of LSCs in the patients with durable TFR. However, CTLs against other antigens, such as PRAME or PR3,^19^ would also be associated with the maintenance of CMR as there were some TFR patients without CXorf48-specific T cells.

As TKIs including imatinib are known to suppress patients’ immune cells,^20, 21^ it is possible that patients’ immune systems effectively control the remaining LSCs especially after withdrawal of TKIs, and this phenomenon might occur predominantly in patients with durable TFR. For example, Mizoguchi *et al.* have shown that the percentage of NK cells is higher in patients with sustained CMR after imatinib discontinuation.^22^ Therefore, immunotherapy enhancing immunity against LSCs is a promising treatment option for achieving TFR in CML patients. Stem cell transplantation, which is one of the established forms of cellular immunotherapy, has resulted in a cure for a majority of CML patients.^23, 24^ However, the details of T-cell immunity against CML-LSC in a TFR-setting have not been fully described. Here, we demonstrated the potential role of T-cell immunity against CXorf48 antigen in CML patients who achieved TFR and suggested this antigen could be a valuable therapeutic target in CML patients.

Promising results for several checkpoint inhibitors have been reported in various cancers including melanoma, lung cancer and Hodgkin lymphoma.^25, 26, 27, 28^ In these malignancies, somatic mutations resulting in amino acid changes give rise to new epitopes recognized by CTLs, called neoantigens.^29, 30^ However, the *BCR-ABL1* driver mutation alone can cause leukemia, and non-driver mutations are limited in CML.^31^ Thus, checkpoint inhibitors alone may not be sufficient to evoke effective immunity against CML-LSCs. Instead, overexpressed antigen, such as CXorf48, may be useful for eradicating CML-LSCs.

One prospective strategy for obtaining TFR would be to maintain CMR by using TKIs for several years, then stop TKI treatment and start immunotherapy against LSC antigens including CXorf48. Such immunotherapies may include vaccination therapy or adoptive T-cell therapy using T cells transduced with antigen-specific T-cell receptor genes.

In conclusion, we identified the epitope peptide derived from the CML-LSC antigen CXorf48. CTLs induced by this peptide recognized LSCs from CML patients. Additionally, CXorf48-specific T cells were observed only in patients who had achieved TFR. Our data strongly suggest that augmentation of the response against LSC antigen by immunotherapy may be useful for achieving TFR after TKI withdrawal in patients with CML. While further clinical study monitoring CXorf48-specific CTLs after withdrawal of TKIs with a larger number of CML patients is necessary, the present data provide strong biological evidence to initiate clinical trials.

## Figures and Tables

**Table 1:** Detection of anti-CXorf48 CTL in CML patients after cessation of imatinib

*Patient no.*	*Anti-CXorf48 CTL*	*Clinical course*	*Time of relapse (months)*	*Observation period (months)*
1	−	Molecular relapse	3	70
2	−	CMR	no	68
3	−	Molecular relapse	3	63
4	−	CMR	no	61
5	+	CMR	no	61
6	−	CMR	no	56
7	−	Molecular relapse	3	51
8	−	CMR	no	46
9	−	Molecular relapse	5	38
10	−	Molecular relapse	2	38
11	−	Molecular relapse	4	38
12	+	CMR	no	37
13	+	CMR	no	37
14	−	Molecular relapse	7	29

Abbreviations: CML, chronic myeloid leukemia; CMR, complete molecular response; CTL, cytotoxic T cell.
