# Supplementary material for: CXorf48 is a potential therapeutic target for achieving treatment-free remission in CML patients
Source: Blood Cancer J. 2017 Sep 1;7(9):e601–. doi: 10.1038/bcj.2017.84 (PMC5709753; doi:10.1038/bcj.2017.84)
Supplement: Supplementary Tables [file bcj201784x1.docx]

**Supplementary files**

Supplementary Table 1

Candidate peptides for HLA-A*24:02-binding epitopes from CXorf48

Word file

Supplementary Table 2

Relationship between anti-CXorf48 CTLs and TFR

Word file

**Supplementary Table 1. Candidate peptides for HLA-A*24:02-binding epitopes from CXorf48**

| Start position | Subsequence | BIMAS score | SYFPEITHI score |
| --- | --- | --- | --- |
| 126 | IYISNSIYF | 150,000 | 22 |
| 208 | GYVPQVDDI | 90,000 | 22 |
| 49 | DYGMIDESI | 70,000 | 22 |
| 142 | DFVPYKGDL | 36,000 | 17 |
| 197 | FFTLDSVKL | 22,000 | 18 |

**Supplementary Table 2. Relationship between anti-CXorf48 CTLs and TFR**

|  | anti-CXorf48 CTLs(-) | anti-CXorf48 CTLs(+) |
| --- | --- | --- |
| TFR | 4 | 3 |
| non-TFR | 7 | 0 |
|  | 11 | 3 |
